# ZEPPI: proteome-scale sequence-based evaluation of protein-protein interaction models

**DOI:** 10.21203/rs.3.rs-3289791/v1

**Published:** 2023-09-18

**Authors:** Haiqing Zhao, Diana Murray, Donald Petrey, Barry Honig

**Affiliations:** 1 Department of Systems Biology, Columbia University Irving Medical Center, New York, NY 10032, USA; 2 Department of Biochemistry and Molecular Biophysics, Columbia University Irving Medical Center, New York, NY 10032, USA; 3 Department of Medicine, Columbia University, New York, NY 10032, USA; 4 Zuckerman Mind Brain and Behavior Institute, Columbia University, New York, NY 10027, USA

**Keywords:** Protein-protein interactions, protein structure, coevolution, protein complex prediction

## Abstract

We introduce ZEPPI (Z-score Evaluation of Protein-Protein Interfaces), a framework to evaluate structural models of a complex based on sequence co-evolution and conservation involving residues in protein-protein interfaces. The ZEPPI score is calculated by comparing metrics for an interface to those obtained from randomly chosen residues. Since contacting residues are defined by the structural model, this obviates the need to account for indirect interactions. Further, although ZEPPI relies on species-paired multiple sequence alignments, its focus on interfacial residues allows it to leverage quite shallow alignments. ZEPPI performance is evaluated through applications to experimentally determined complexes and to decoys from the CASP-CAPRI experiment. ZEPPI can be implemented on a proteome-wide scale as evidenced by calculations on millions of structural models of dimeric complexes in the *E. coli* and human interactomes found in the PrePPI database. A number of examples that illustrate how these tools can yield novel functional hypotheses are provided.

## Introduction

The past decade has seen continuing developments in the prediction of protein-protein interactions (PPIs). One can trace these advances to the use of amino acid coevolution to predict inter-residue contacts^1,2^. These methods have been used to predict protein structures^3–5^ and, more recently, to predict interaction partners and interfacial residues involved in PPIs^6–9^. The underlying premise is that functional interactions between two residues will result in their coevolution, which should be reflected in species-paired multiple sequence alignments (MSAs) of putative orthologues and detectable through mutual information (MI) based metrics between the two positions in the alignment. A complication is that the correlation between two residue positions *i* and *j*, i.e., two columns in the MSA, may result from an indirect coupling of *i* and *j* through their interaction with a third residue *k*. To solve this problem, methods such as Direct Coupling Analysis (DCA)^3,10^, sparse inverse covariance (PSICOV)^11^, EVcouplings^6,8^, and Gremlin^4,7^, have been developed. However, these methods rely on the availability of deep MSAs and thus have almost exclusively been applied to bacterial systems. In contrast, as we demonstrate below, ZEPPI can be applied on a genome-wide scale to eukaryotic proteomes with relatively shallow MSAs.

AlphaFold-Multimer^12^ (AFM) has fundamentally changed the landscape of the prediction of structures of multi-protein complexes. There have been continuing improvements in AFM-based methods^13,14^ as is evident from the substantial progress in the recent CASP-CAPRI^15,16^ experiment^17^. An underlying problem for MSA-based methods is that, for a heterodimeric pair, it is generally necessary to carry out a species-based matching of the two query sequences which limits application to eukaryotic organisms due to the relatively limited number of sequences available for a paired MSA. Recently, RoseTTAFold/AlphaFold, was used to screen 4.3 million potential yeast PPIs with alignments containing >200 sequences and proteins with < 1500 amino acids per pair^18^. This was enabled in part by the large number of fungal genomes available but, as the authors point out, by the extensive amount of structural information embedded in RoseTTAFold^18^. However, applying deep learning to predict whether and how two proteins interact for entire eukaryotic proteomes remains computationally challenging.

Docking-based methods predict models of protein dimers based on the structures of the constituent monomers^19–22^ but have not been applied on a proteome-wide scale or to predict *whether* two proteins interact. Template-based modeling^23^ is an alternate approach where the structures of individual proteins are superimposed on structurally similar proteins that appear in a complex present in the PDB^24^. In a series of papers, we reported the PrePPI (Predicting Protein-Protein Interactions) algorithm and database^25–27^ that rely on template-based modeling and, through a highly efficient scoring function, leverage structural information on a truly proteome-wide scale. For example, PrePPI effectively screens the ~200 million possible pairwise combinations of human proteins which, in practice, amounts to billions of possible interactions among full-length proteins and protein domains. Based on a false positive rate of <0.005, 1.3 million high confidence predictions appear in the PrePPI online database with 370K corresponding to direct binary interactions ^27^.

Here we use PrePPI predicted complexes in the *E. coli* and human proteomes to examine the extent to which simple evolution-based metrics are informative even in those cases for which the multiple sequence alignment (MSA) depth is shallow. Once an interface is defined, we expect that MI calculations alone would be sufficient, even for eukaryotic proteins, as the deep MSAs required for DCA analysis would no longer be necessary. Our method, ZEPPI (for Z-score Evaluation of Protein-Protein Interfaces), uses paired MSAs to determine coevolutionary information across interfaces but also leverages sequence conservation which provides an additional signal as to the reliability of a predicted interface. An essential feature of ZEPPI is the comparison of evolutionary metrics derived from MSA positions corresponding to residues in predicted interfaces versus positions corresponding to randomly chosen residues.

Our focus on interfacial residues leads to a significant speedup in the evaluation of dimeric complexes that allows us to apply ZEPPI on a proteome-wide scale. Similarly, our finding that DCA is not needed for evaluating heterodimeric complexes effectively removes the need for deep species-paired MSAs. As shown below ZEPPI is extremely effective in distinguishing correct from incorrect protein-protein interfaces as indicated by tests on PDB structures and on a CASP-CAPRI benchmark set^15,28^. Most notably there is a strong inverse correlation between ZEPPI scores and false positive rates (FPRs) for PrePPI predictions thus providing strong support for the reliability of ZEPPI’s efficacy and applicability to proteome-wide interactomes. We use a combined PrePPI/ZEPPI screen to identify a large number of novel interactions that do not appear in any database. A number of examples are discussed below.

## Results

### ZEPPI overview

**Figure 1.**
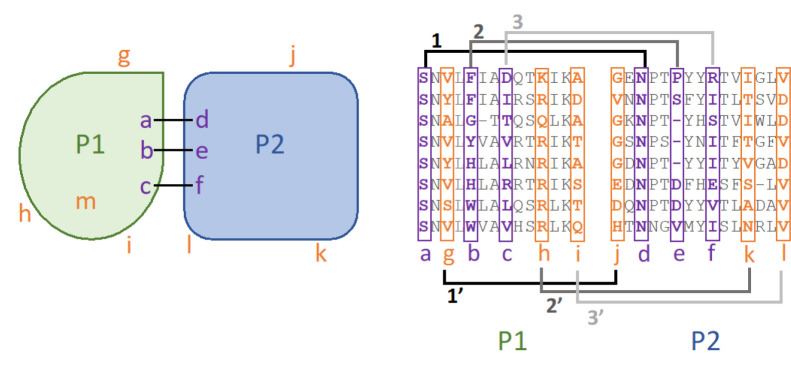
Schematic of the ZEPPI algorithm. Two proteins, P1 and P2, form a complex with three interfacial contacts between residues **a** and **d**, **b** and **e**, and **c** and **f** (purple; left), respectively labeled as **1**, **2**, **3** in the right panel. Various evolutionary metrics (see text) are calculated from the corresponding columns in the paired alignment (purple; right). “Fake interfacial contacts” are generated between randomly chosen surface residues outside the interface, shown by **1’**, **2’** and **3’** on the right panel. When the number of surface residues outside the interface does not exceed the number of interfacial residues, buried residues (**m**) are considered as well. The same metrics are calculated from the corresponding columns in the MSA (orange; right) for each of 100 samples.

Figure 1 summarizes the ZEPPI algorithm. The procedure starts with a structural model of a complex between proteins P1 and P2 (left panel) and a paired MSA (right panel). Contacting interfacial residues are identified; in this case, P1-a contacts P2-d, P1-b contacts P2-e, and P1-c contacts P2-f. A paired MSA is created and used to calculate the following metrics for each of the four interfacial contacts as described in Methods: mutual information (MI), conservation (Con), direct coupling (DCA), and average product corrected (APC)^29^ metric for each. The resulting values are then averaged over the interfacial contacts yielding 6 metrics. in addition, the highest single-contact score for each metric, denoted as “top”, is retained, resulting in a total of twelve metrics that characterize a predicted interface. Columns in the MSA corresponding to contacting interfacial residues are colored in purple. For example, the residues in columns P1-a and P2-d are almost completely conserved and would give a strong Con signal but a weak MI signal. Columns P1-b and P2-e show no obvious Con or MI signal but P1-c and P2-f show a clear MI signal.

The next step is to carry out the same procedure for a set of randomly chosen surface residues that are not in the interface. These are denoted in orange and are treated as if they were interfacial so that, for example, the metrics calculated between columns P1-a and P2-d are replaced by those between P1-g and P2-j. Each contact in the real interface is replaced in this way by fake contacts as indicated in the figure. Note that when the number of surface residues outside the interface is less than the number of residues in the interface, buried residues, e.g. P1-m, are included in sampling. This occurs for < 10% of PPIs evaluated. A hundred fake interfaces with corresponding values for the twelve metrics are generated in this way. A Z-score for the predicted interface is then calculated for each of the metrics based on the values for the real interface as compared to the values obtained for the 100 fake interfaces. In practice, based on results for PDB complexes (see next section) DCA is only used for homodimers where it makes an important contribution and where the computational cost is small given the relatively small number of homodimers in a proteome.

### Testing ZEPPI on PDB complexes

Dimeric PDB complexes were collected from the first bioassembly, as defined in the PDB structure file, for both bacterial and human complexes^24^. We tested performance with both prokaryotic and eukaryotic proteomes which, overall, have very different MSA depths. As described in Methods, complexes were selected based on resolution, chain length and the requirement that the proteins in the complex are from the same species. In total, 279 bacterial heterodimers, 247 human heterodimers, 3976 bacterial homodimers, and 977 human homodimers, for a total of 5,479 dimer structures, were obtained. For each complex we calculated the 12 metrics.

**Figure 2.**
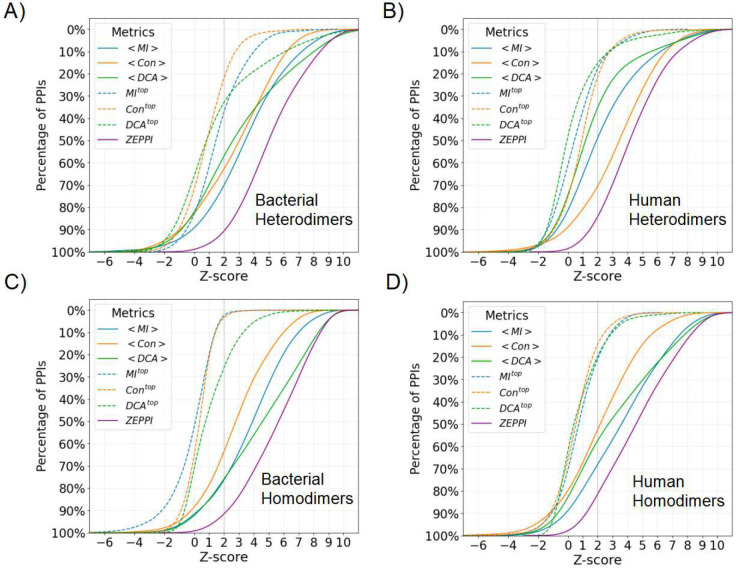
Percentage of PDB PPIs as a function of Z-score. Colors and line types in the legend indicate curves for different metrics each of which corresponds to the maximum of the raw and APC values for a given PPI. The mean and top metric of all interface contacts are denoted as <>, and ^top^, respectively. The ZEPPI score for a given PPI is the largest Z-score among all metrics and the curve for all PPIs is shown in purple. See Supplementary File 1.

Figure S1 and S2 plot, for each of the twelve metrics, the fraction of PPIs with a Z-score above the threshold denoted along the x-axis. For example, in Figure S1A, at a Z-score of 2, the metric for average APC-corrected MI, $<{MI}_{APC}>$, by itself recovers 60% of bacterial heterodimers whereas integrating all metrics (Figure 2) recovers 80%. Overall, it is evident that, for heterodimers, the APC correction improves performance relative to raw (uncorrected) metrics for MI and DCA but not for Con (Figure S1, A and B). In contrast, for homodimers, the APC corrected Con metric is more effective than the corresponding raw metric (Figure S1, C and D). Further, choosing the top value for each metric is less effective than choosing the value averaged over the entire interface (dashed curves versus solid curves, Figures S1–S2). This is not unexpected since all contacts identified in PDB complexes are presumed to be correct and likely contribute to the total score. However, this is not necessarily the case with docked and predicted complexes as depicted below.

Figure 2 contains similar plots to those reported in Figures S1 and S2 but, for a given metric, the higher value of raw versus APC-corrected metric is chosen for each complex. The ZEPPI curve (purple) is generated by choosing the metric with the highest score for each complex. For the remainder of the paper, the ZEPPI score for a given complex corresponds to the maximum value from among the complex’s 12 metrics.

Figure 2 contains an evaluation of ZEPPI on PDB complexes. Of course, in all cases ZEPPI outperforms any individual metric since it “chooses” the best performing metric for each complex. For bacterial heterodimers (Figure 2A) MI is the best performing metric although DCA is slightly better at high Z-scores and Con performance is similar to both MI and DCA. In contrast, Con is clearly the most important metric for human heterodimers. We suggest that the difference between human and bacteria is the greater coevolutionary divergence underlying bacterial MSAs as opposed to eukaryotic MSAs. Of note, the overall ZEPPI performance is very similar for bacteria and human with, in both cases, about 65% of the complexes having a ZEPPI score > 4.

For homodimers, the two coevolutionary metrics perform the best with DCA performing better than MI at high Z-values for bacteria while MI is, overall, the best performer for human. The improved performance of coevolution for homodimers is likely due to the fact that MSA sequence depth for homodimeric complexes is much larger (reflecting two copies of a single protein) than for heterodimers. It is interesting that in all cases, MI performance is comparable to or better than that of DCA, except for bacterial homodimers which are associated with the deepest MSAs. But even in that case the differences manifest only at high Z-scores. Most importantly, since DCA contributes very little for human complexes and, given its need for deep MSAs and the extra computer time required in its use, below we only use DCA for homodimers. Of note, Bitbol has reported that, using an iterative pairing algorithm, MI alone performs at least as well as DCA in the sequence-based identification of protein-protein interaction partners^9^.

#### Effect of MSA depth –

**Figure 3.**
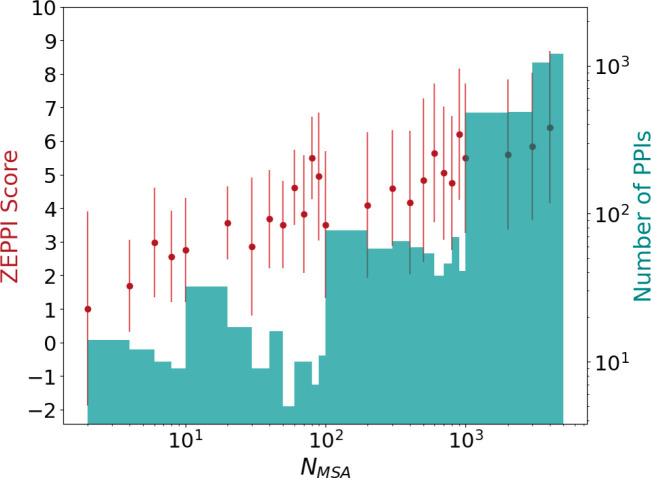
Effect of MSA depth on ZEPPI score for PDB dimers. The ZEPPI score is plotted against MSA depth, N_MSA_, where each red dot and error bar correspond to the average and standard deviation of the ZEPPI score for the PPIs in a given bin of N_MSA_ values. A histogram of the numbers of PPIs in each bin is shown in green. Data are plotted on a log scale for N_MSA_ and the number of PPIs. N_MSA_ is the depth of the paired MSAs after checking the coverage of surface residues (see Methods and Supplementary File 1.).

Figure 3 plots ZEPPI score (red dots) versus the sequence depth of the MSAs (N_MSA_). The histogram (green) displays the number of interfaces as a function of N_MSA_. On average, ZEPPI score is seen to increase with increasing MSA depth although there are examples where ZEPPI scores > 2 are obtained for very shallow MSA depths (N_MSA_ < 10). Most of these result from significant sequence conservation of interfacial residues but there are cases where even MI yields a significant signal. Although these few cases may well be statistical anomalies, there are many high-scoring interfaces of relatively shallow depths with values of N_MSA_ in the range of 10 to 100. Note that N_MSA_ here is a raw number that does not include the low-weighting of redundant sequences. It is generally accepted that, for most applications, N_MSA_ should be at least the sum of the number of residues in each protein^7,30–32^ and is typically taken to be greater than 500 or 1000 for predictions of protein-protein interactions^7,31^. Our results highlight the success of ZEPPI in leveraging even shallow MSAs, made possible by the evaluation of interfacial residues in experimentally determined structures.

### Test on CAPRI benchmark decoys

We tested the performance of ZEPPI in differentiating good versus poor models in a widely used decoy set, score_set^28^, which was derived from targets from the CASP-CAPRI experiment. The score_set contains docking models predicted by 47 different groups for proteins from bacteria, yeast, vertebrates and artificial design. We considered 13 widely studied targets which, overall, have 18,538 corresponding decoys: 10% represent docking predictions of acceptable, medium or high quality based on CAPRI-defined criteria. We combine these three categories and annotate the group as “acceptable+”, whereas the remaining 90% are annotated as “incorrect.” Even though two of the targets, T53 and T54 contain designed proteins, they both have MSAs with N_MSA_ values of 2110 and 198, respectively. Table S1 reports MSA depth for all targets along with the number of acceptable+ and incorrect decoys, and the area under the ROC curve (AUROC) for each target. In contrast to the results with PDB complexes, the top metrics contribute to the ZEPPI score to a greater extent likely because the interfaces for acceptable+ models have inaccuracies. It is clear from the table that shallow MSA depths (<100) can produce good AUROCs, particularly for T47 which has an AUROC of 0.93 and N_MSA_ of only 24.

Figure S3 plots the percentage of all models that have a given ZEPPI score in each of the four categories across targets. There is a clear distinction between acceptable+ and incorrect decoys with essentially 90% of the acceptable+ models having Z-scores > 2. Nevertheless, some incorrect decoys do have high Z-scores and some correct decoys have low Z-scores. For example, T40 (Table S1, AUROC = 0.66) is derived from a trimeric complex between a bovine protein and two copies of the same plant protein that bind in different locations. Only one interface is considered in the decoy set but the other forms a second interface complicating the sampling of non-interacting surface residues in creating fake interfaces. This issue that does not affect docking algorithm performance but compromises ZEPPI performance.

Table 1 compares ZEPPI performance across targets to that of other methods^33–38^, most of which are based on deep learning. The data for other methods was taken from Table S8 of Réau *et al.*^34^ (see also Methods). ZEPPI, despite not involving training, is essentially tied as the top performer as measured by AUROC and is the best performer based on top 100 Success Rate. However, ZEPPI is outperformed by a number of other methods as measured by top 1 and top 5 Success Rates. Based on these criteria, iScore is the best performer. Of note, AUROC is affected by the distribution of false and true positives in a list of predictions while Success Rate depends on the number of good predictions at the top of the list. Success Rates are central to CASP-CAPRI rankings while ROC curve performance may be more important in asking whether a particular prediction is correct.

### Evaluating PrePPI PPI models with ZEPPI

In recent work we reported PrePPI calculations for the human and *E. coli* interactomes represented by models for the full-length sequences and constituent domains^27^. A structural modeling score, SM, was trained on the HINT high-quality literature-curated (HINT-HQ-LC) dataset for human PPIs. HINT-HQ-LC datasets are designed to contain high confidence binary interactions^39^. ROC curves were reported for testing the PrePPI human and *E. coli* models on the human and *E. coli* HINT-HQ-LC datasets using 10-fold cross-validation. These yielded AUROC values of 0.83 and 0.88, respectively, thus, attesting to the overall high-quality of the predictions.

Figure S4 displays violin plots for the range of ZEPPI scores for PrePPI predictions in different bins of false positive rates (FPR). For PrePPI predictions of higher confidence (lower FPR), the median ZEPPI score is larger. These results provide a strong consistency check in that better structural models as defined by PrePPI produce stronger evolutionary signals as measured by ZEPPI. For bacterial heterodimers (Figure S4A), at FPR < 10^−4^, the percentage of predicted PPIs with a ZEPPI score > 2, 3, 4 is 94%, 81% and 67%, respectively. The comparable numbers for PDB structures (see discussion of Figure 2) are 95%, 85% and 71% suggesting that PrePPI’s highest confidence predictions have ZEPPI scores close to those of PDB structures. Performance deteriorates as FPR increases but there are still many good ZEPPI scores for higher FPR values. The distributions in Figure S4 demonstrate that high and low ZEPPI scores are obtained in all FPR bins suggesting ZEPPI score can be used as an additional evidence source for prioritizing PrePPI models.

### The *E. coli* structural interactome

Table 2A lists the number of *E. coli* PPIs (out of the 5.4 million for which a model can be built) and the number of proteins that comprise these interactions for different FPRs and ZEPPI scores. At FPR < 0.01 PrePPI predicts 71K PPIs involving 3.5K proteins, and these numbers are significantly decreased when more stringent PrePPI FPRs and ZEPPI scores are applied. 2.3K PPIs satisfy the highly restrictive criteria of FPR < 0.0001 and ZEPPI score > 4.

Table 2A also lists the overlap of ZEPPI-filtered PrePPI predictions with PPIs annotated in experimental databases (DBs). Any PPI that appears in the listed databases (see Methods) is considered whether or not the interaction is likely to be direct or indirect so as to determine the number of truly novel PPIs that our methods predict. At the most stringent end of the scale (FPR < 0.0001, ZEPPI score > 4) 518 novel predictions are made. On the other hand, as an example, there are 21,000 novel predictions made for FPR < 0.05 and ZEPPI score > 4 suggesting that using ZEPPI may facilitate the discovery of meaningful predictions that might be missed based on PrePPI alone.

### The human structural interactome

Table 2B presents results for the PrePPI-predicted human interactome that parallel those for *E. coli* (Table 2A). In contrast to PrePPI results reported recently, which are based on both structural and non-structural evidence, Table 2B reports data for SM only, i.e. domain-domain structure-based predictions. A total of 1.3M PPIs are predicted with an FPR <0.01 which is an overly tolerant criterion. This number is reduced to only 130K for FPR <0.001 and only 12K for FPR <0.0001^27^. ZEPPI provides an alternate filter; for example ZEPPI = 4 reduces the number of predictions to 228K, 31K and 7K for FPR <0.01, 0.001 and 0.0001, respectively.

As is the case for *E. coli* (Table 2A), most PrePPI predictions do not appear in any experimental database nor in STRING^40^ which includes many PPIs inferred from sequence relationships (collectively, “PPIs in DBs”). Although PrePPI provides structural models for many experimentally determined interactions, its value is also in hypothesis generation as many of its predictions are novel. At the highest confidence level (FPR <0.0001, ZEPPI>4), there are 2983 novel human PPI predictions. ZEPPI can be used to discriminate predictions at different PrePPI confidence levels, as indicated in the following examples.

### Biological applications of ZEPPI/PrePPI

#### Distinguishing among homologs:

An issue with PrePPI and other PPI prediction methods is that they encounter difficulties in predicting binding specificity when closely related homologs are involved. For example, based on a relatively small number of templates, there are many predicted interactions for the small GTPase K-Ras with GTPases, GAPs, GEFs, and other signaling proteins. Based on the X-ray complex for H-Ras/Grb14^41^ (PDB ID: 4k81), PrePPI makes predictions (FPR ≤ 0.005) for K-Ras interactions with Grb7, Grb11, and Grb14. However, ZEPPI is significant (Z = 3.5) for only KRAS-Grb7^42^, the one for which there is evidence of an interaction (in DBs as defined above). This is a case where the structural models are too similar to be distinguished from one another but where there is a clear sequence signal that ZEPPI detects among interfacial residues.

#### A possible role for K-Ras in synaptic signaling:

**Figure 4.**
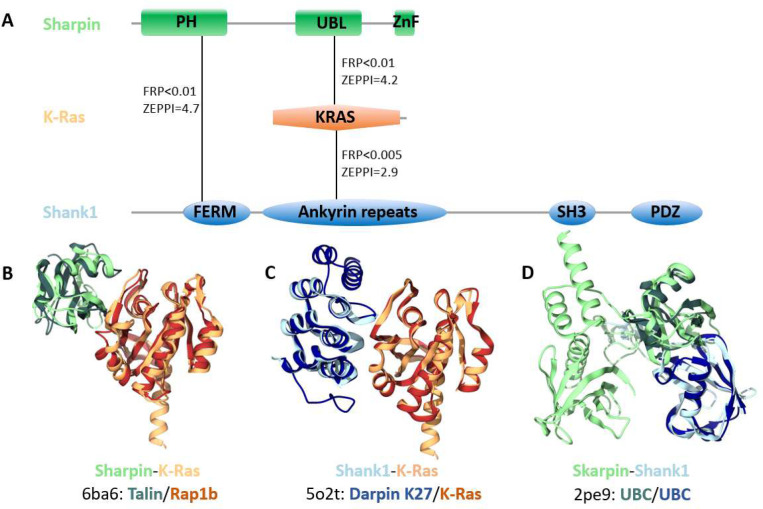
High-confidence PPIs in synaptic signaling. A) PrePPI and ZEPPI predict interactions among Sharpin (the Shank-interacting protein-like 1, colored in green), the small GTPase K-Ras (orange), and Shank1 (the SH3 and multiple ankyrin repeat domains protein 1, colored in blue). Solid lines between protein domains denote the domains involved in the PPIs which are depicted as backbone ribbons. In B-D), the darker colored ribbons represent the chains from the PDB PPI template and are defined below the query protein names: B) Sharpin-K-Ras; C) K-Ras-Shank1; and D) Sharpin-Shank1. In all but two cases (KRAS-6ba6:B and KRAS-5o2t:A), the pairwise sequence identities between the queries and the respective template chains is less than 25%.

As shown in Figure 4A, PrePPI predicts interactions among K-Ras, Sharpin (the Shank-interacting protein-like 1) and Shank1 (the SH3 and multiple ankyrin repeat domains protein 1). Structural predictions are shown for 1) the Sharpin ubiquitin-like (UBL) domain and K-Ras (Figure 4B); 2) K-Ras and Sharpin ankyrin repeats (Figure 4C); and 3) Sharpin PH domain and Shank1 FERM domain (Figure 4D). Sharpin has previously been shown to interact with Shank1 and both co-localize at synaptic sites in mature neurons^43^. Altogether, our predictions (Figure 4) and the related experimental evidence suggest a novel role for K-Ras in synaptic signaling. Indeed, a recent study^44^ found that mutant K-Ras increases synaptic transmission in inhibitory neurons, while it promotes the cell death of excitatory neurons.

#### Secreted peptide fragments in the pancreas:

Chymotrypsin-like elastase family member 1 (CELA1) is a secreted elastase with high pancreatic expression. Recent studies have implicated peptides produced from the amyloid precursor protein (APP) in metabolic diseases^45,46^. In particular, human pancreatic islet cells process APP to release secreted fragments of APP (sAPP). The CELA1-APP model (Figure S5) suggests a pancreatic-specific mechanism for the production of sAPP.

#### Role of Cystatins in tumorigenesis:

Cystatins are inhibitors of cysteine peptidases. In tumor development and cancer progression the balance between cystatins and cysteine peptidases may be disrupted^47^. Cathepsin F (CTSF) was observed to have an anti-tumor effect in lung adenocarcinoma (LUAD)^48^ whereas Cystatin-SN (CST1) promotes the epithelial-mesenchymal transition in LUAD cells^49^. The CST1-CTSF model (Figure S6) suggests that the mechanism of action of CST1, which is highly expressed in LUAD^49^, may be to inhibit the anti-tumorigenic activity of CTSF.

## Discussion

Here we have introduced ZEPPI, a novel method that uses species-paired MSAs as a basis for scoring predicted models of protein-protein interfaces. ZEPPI’s central feature involves the analysis of evolutionary information involving only contacting residues in a 3D structural model. The relatively limited number of residues to be analyzed results in a major reduction in computer time required to evaluate a model. Moreover, ZEPPI extracts signals from shallow MSAs enabled in part by its reliance on sequence conservation as well as mutual information. Deep learning methods implicitly leverage both sources of information but since most analyze entire sequences they are more computationally intensive.

In addition to validation on crystal structures, ZEPPI was tested on thirteen CASP-CAPRI targets and its performance was found to be comparable to or better than other interface evaluation approaches. We note that evolutionary information has been used for some time in the evaluation of docking models but generally in combination with other evidence sources, such as statistical propensities for surface residues to be in protein interfaces. ZEPPI differs from these approaches in its combined use of mutual information and conservation within interfaces and, especially, in its method of calculating Z-scores through the comparison of metrics for positions in the MSAs corresponding to interfacial residues versus positions in the MSAs corresponding to randomly chosen residues outside an interface. Our results on both PDB and CASP-CAPRI complexes demonstrate that ZEPPI provides a computationally efficient and highly effective measure of interface quality that can easily be combined with other sources of evidence.

To demonstrate its computational efficiency, we have applied ZEPPI to 5.4 million *E. coli* PPI interfaces and to 6.2 million (FPR <0.05) human PPI interfaces predicted by PrePPI. As suggested by the results in Table 2, filtering PrePPI predictions by ZEPPI scores has the potential to increase the reliability of high confidence predictions while identifying low confidence PrePPI predictions that are worthy of further consideration. An immediate application of ZEPPI is its integration into the PrePPI algorithm with the goal of combining evolutionary signals with a method based entirely on 3D structure. The integration should prove to be quite valuable, especially in applications to the human proteome and other eukaryotic organisms where available sequence information supports alignments of relatively shallow depth.

The vignettes provided above indicate the ability of ZEPPI to aid in the discovery of novel and potentially important functional hypotheses. In this regard, ZEPPI/PrePPI can be viewed as a hypothesis generating method that could be followed up with slower structure prediction methods ranging from docking to AF-multimer to methods based on their combination as evidenced from the most recent CASP-CAPRI experiment^17^. Of course, alternatively, ZEPPI can be used independent of PrePPI to evaluate any predicted model of a complex. For example, it could be used to distinguish direct physical from indirect interactions in experimentally determined multi-protein complexes.

The combination of PrePPI with ZEPPI suggests a general approach to the proteome-wide prediction of whether and how two proteins interact. ZEPPI can be used to score PrePPI models and thus provide an independent metric for their accuracy. In addition, if one is interested in evaluating specific PPIs, predicted for example by deep learning or docking-based methods, the post-prediction application of ZEPPI would appear to offer an efficient and accurate option. However, in genome-wide applications, we suggest using structure-based approaches to provide interactome-wide yes/no answers along with 3D models and then turning to increasingly accurate deep learning methods for a more limited set of interactions of particular interest.

## Methods

### Selecting bacterial and human PDB dimer structures

Taxonomy and UniProtKB summary files for all PDB chains were downloaded from the Structure Integration with Function, Taxonomy and Sequence (SIFTS) project^50^. From the SIFTS PDB chain taxonomy file, PDB chains that correspond to only one taxonomy ID were selected and then filtered to bacterial and human PDB chains, respectively. The taxonomy list of bacteria was collected from searching both the UniProt proteome^51^ and the NCBI Taxonomy databases^52^. The union of the two searches provided 521,897 unique bacteria taxonomy IDs.

From the SIFTS PDB chain UniProt file, PDB files with only two UniProt IDs for heterodimers and one UniProt ID for homodimers with both chains longer than 30 amino acids are selected. PDBs that have any single chain mapped to ≥ 2 UniProt IDs are excluded to avoid fusion or chimera proteins. Structure resolution information is obtained through the PDB API service^24^. PDBs that are protein-only as the polymer entity type, and either from X-ray with resolution ≤ 4 Å or from EM with resolution ≤ 4.5 Å are selected. NMR structures are not used. Further, through reading the PDB file header, PDBs where the oligomer state of the first BioAssembly (BIOMOLECULE annotations) defined as “DIMERIC” by either the author or software with resolved sequence lengths longer than 30 amino acids are selected. Different PDB dimer structures for the same UniProt ID pairs are collapsed by keeping the structures with better structural resolution or longer chain-concatenated length (at least twice as long). Lastly, to remove closely related homologous protein pairs, we compared the pairwise sequence identities and removed sequence redundant structures where both protein sequences have 90% sequence identity with another structure. The detailed pipeline is provided in the supplemental information.

### Defining protein surface and protein-protein interface

The accessible surface area (ASA) of residues for individual chains A and B, and their complex AB are obtained using our in-house program of surfv^53^. An interface is defined as long as the buried ASA is larger than zero. The interface between proteins A and B consists of contacting residues where the distance between any heavy atoms is less than 6.0 Å. All the residue indices from the PDB are updated after mapping the PDB sequences to their full UniProt sequences using hhalign of the hh-suite package^54^.

### Generating random protein-protein interfaces

The positions of interface residues on proteins A and B in the concatenated MSA are replaced, one by one, with positions for randomly chosen surface residues of the same protein as indicated in Figure 1. If one protein has more interface residues than surface residues that are not on the interface, the sampling pool goes to the entire protein sequence. To ensure statistical significance of the Z-score calculations, 100 random interfaces are generated for each protein-protein interface.

### Generating and pairing MSAs

To avoid biased sequence sampling due to over-studied model species, we carried out homolog sequence search on 5,090 representative proteomes that were carefully curated and selected in EggNog 5.0^55^. This database includes 4,445 prokaryotic reference genomes selected from original 25,038 bacteria genomes, and 477 eukaryotic genomes. Homologous sequences are searched using Jackhmmer (hmmer-3.2.1)^56^ with 5 iterations and the default E-value of 0.001. In the final outputted multiple sequence alignment, only the sequence with highest identity to the query is kept as the representative sequence for each species.

The MSAs of two proteins, p1 and p2, are paired based on the shared common species. Sequence rows that cover less than 50% of surface residue positions of p1 or p2 are excluded from the paired MSA. MSA columns, either for interface residue or surface residue positions, that have more than 50% gaps are excluded.

### Calculating mutual information, conservation, DCA, and their APC-corrected terms

For two positions (a,b) in the paired MSA, their mutual information (MI) is calculated through Eq. 1, where x and y denote their amino acid type and the gap is treated as the 21st amino acid type or state. The p(x) and p(y) are the frequencies of a certain amino acid type and p(x,y) is the frequency of a pair of different amino acid types. The conservation score between two positions (a,b) is defined through the complement of their normalized joint entropy S(a,b) (Eq. 2). The direct coupling information is calculated through the mean field DCA method which is based on the maximum-entropy model^3^. The final direct coupling information is quantified using a similar definition as in the mutual information except $P^{(dir)}(x,y)$ involves only the isolated direct coupling strength of (a,b) from the DCA calculations (Eq. 3).

(Eq. 1) $$MI(a,b)=-\sum_{x\in \{1..21\}}\sum_{y\in \{1..21\}}p(x,y)ln\frac{p(x,y)}{p(x)p(y)}$$

(Eq. 2) $$Con(a,b)=1-\frac{S(a,b)}{2*log21}$$

(Eq. 3) $$DI\left(a,b\right)=-\sum_{x\in \left\{1..21\right\}}\sum_{x,y\in \left\{1.21\right\}}P^{\left(dir\right)}\left(x,y\right)ln\frac{P^{\left(dir\right)}\left(x,y\right)}{p\left(x\right)p\left(y\right)}$$

The average product correction (APC) is applied to measurements as denoted throughout the text. Taking MI as an example, the APC term between position (a,b) (from p1 and p2, respectively) is calculated as the product of the average MI signal of position a with positions in p2, and position b with positions in p1, between interfacial residues on both proteins, then normalized by the average measurement of all protein to the other (Eq. 4). The APC-corrected MI is given by Eq. 5, and the same correction correspondingly applies to Con, and DCA scores.

(Eq. 4) $$APC_{MI}\left(a,b\right)=\frac{<MI\left(a,\;y\right)>\text{*<}MI\left(b,\;x\right)>}{<MI\left(x,y\right)>}$$

(Eq. 5) $$MI_{APC}\left(a,b\right)=MI\left(a,b\right)-APC_{MI}\left(a,b\right)$$

### Calculating Z-scores of the interface

For each interface contact of a given interface between protein p1 and p2 and the generated 100 random interfaces, the following six measurements are calculated: mutual information, conservation, direct coupling information and their corresponding APC-corrected terms. Of all the interface contacts, we choose the top and the mean as the representative metric for each measurement, denoted as ${MI}^{\text{top}}$ and <MI>, for example. The Z-score of the 12 metrics are then calculated for the given interface versus the generated random interfaces. The larger Z-score of the raw metric versus its APC-corrected metric is taken as the Z-score for this metric. The maximum of all metrics is taken as the final ZEPPI score.

### Building the *E. coli* experimental PPI database

The experimental database of *E. coli* PPIs is integrated from several major resources including Interactome3D^57^, HINT^39^, APID^58^, STRING^40^ and Ecocyc^59^, as well as previously known large-scale *E. coli* PPI high-throughput screening with experimental methods such as APMS^60^ and Y2H^61^. Another well-known experimental database BioGrid^62^ is not included due to the lack of *E. coli* (NCBI Taxonomy ID: 83333) PPIs included. Before their integration, each database was pre-processed by selecting only *E. coli* K12 proteins (proteome size: 4391) and sorting the uniport IDs for each pair of PPIs. During the integration, redundant PPIs were removed. Note that Interactome3D also includes homology-modeled PPIs and the STRING database has many inferred PPIs, which are not determined by direct physical interaction experiments but inferred by other methods such as gene-related methods or species PPI transfer. By excluding these two contributions, we also built a purely experimental PPI database of *E. coli* based on direct physical experiments. In all, there are 565,007 PPIs in the integrated experimental database set and 45,634 PPIs in the physical experimental PPI dataset.

In summary, the integrated experimental database set includes: all HINT binary and complex PPIs (updates of 2021/11), all APID PPIs (updates of 2021/11), all Interactome3D PPIs (updates of 2021/11), all STRING PPIs (v11.5), the gold standard dataset used in Zhang and coworkers’ Threpp work^63^, the high throughput experimental PPI set from Threpp (Table S1), the EcoCyc PPIs downloaded Cong *et al*.^64^(Table S5), the Y2H PPI set from Rajagopala *et al*.^61^ (Supplementary Table 2), the high-confidence and median-confidence APMS PPI set from Babu *et al*.^60^ (Supplementary Table 2). For the physical experimental PPI dataset, only physical links in the STRING database with experimental score >0 are included; only the PDB subset of Interactome3D is included; the other datasets remain the same as in the integrated experimental database.

### Building the human experimental PPI database

The integrated human experimental PPI database consists of the following resources: all HINT binary and complex PPIs, all Interactome3D PPIs, all APID PPIs, all STRING PPIs (v11.5), all BioGrid^62^ PPIs, all HURI^43^ PPIs, and the HC-2016 set from PrePPI^26^. In total, there are 6,068,248 PPIs collected from the above-mentioned experimental databases with the large majority derived from STRING.

## Supplementary Material

Supplement 1

## Figures and Tables

**Table 1.** Performance of different scoring methods on CAPRI decoys. AUROC in this table is averaged over values for each of 13 targets. Success Rates of Top N indicates the number of targets where there are acceptable or better predictions in the Top N predictions.

Method	AUROC	Success Rates		
		Top1	Top5	Top100
ZEPPI	0.72 ± 0.13	2/13	2/13	12/13
HADDOCK	0.57 ± 0.23	2/13	3/13	9/13
iScore	0.68 ± 0.21	5/13	6/13	9/13
DeepRank	0.64 ± 0.19	1/13	1/13	9/13
DOVE	0.56 ± 0.14	1/13	2/13	10/13
GNN-DOVE	0.63 ± 0.16	1/13	6/13	8/13
DeepRank-GNN	0.72 ± 0.19	1/13	5/13	10/13

**Table 2.** Number of proteins, PPIs and novel predictions for different combinations of PrePPI FPRs and ZEPPI-scores for *E coli*. and Human.

**A). *E coli***								
**PrePPI FPR (≤)**	*0.05*		*0.01*		*0.001*		*0.0001*	
**ZEPPI Score (≥)**	−	*4*	−	*4*	−	*4*	−	*4*
**#PPIs**	303,212	38,062	71,151	17,098	10,336	6,002	3,151	2,355
**# Proteins**	3,941	3,671	3,557	3,030	2,464	1,993	1,605	1,350
**# PPIs in DBs**	38,916	9,069	14,580	6,090	5,289	3,528	2,386	1,837
**# Novel PPIs**	264,296	28,993	56,571	11,008	5,047	2,474	765	518
**B). Human**								
**PrePPI FPR (≤)**	*0.05*		*0.01*		*0.001*		*0.0001*	
**ZEPPI Score (≥)**	–	*4*	–	*4*	–	*4*	–	*4*
**#PPIs**	6,209,528	1,002,052	1,271,323	228,321	130,447	30,572	11,896	7,392
**# Proteins**	16,780	15,987	13,903	11,781	6,358	5,250	2,882	2,441
**# PPIs in DBs**	463,971	130,426	148,461	54,131	20,605	10,762	6,293	4,409
**# Novel PPIs**	5,745,557	871,626	1,122,862	174,190	109,842	19,810	5,603	2,983

See Supplementary Files 2–4.
