# Supplementary material for: ZEPPI: proteome-scale sequence-based evaluation of protein-protein interaction models
Source: Res Sq. 2023 Sep 18:rs.3.rs-3289791. Preprint. [Version 1] doi: 10.21203/rs.3.rs-3289791/v1 (PMC10543297; doi:10.21203/rs.3.rs-3289791/v1)
Supplement: Supplement 1 [file NIHPPRS3289791V1-supplement-1.pdf]

Supplemental Information:

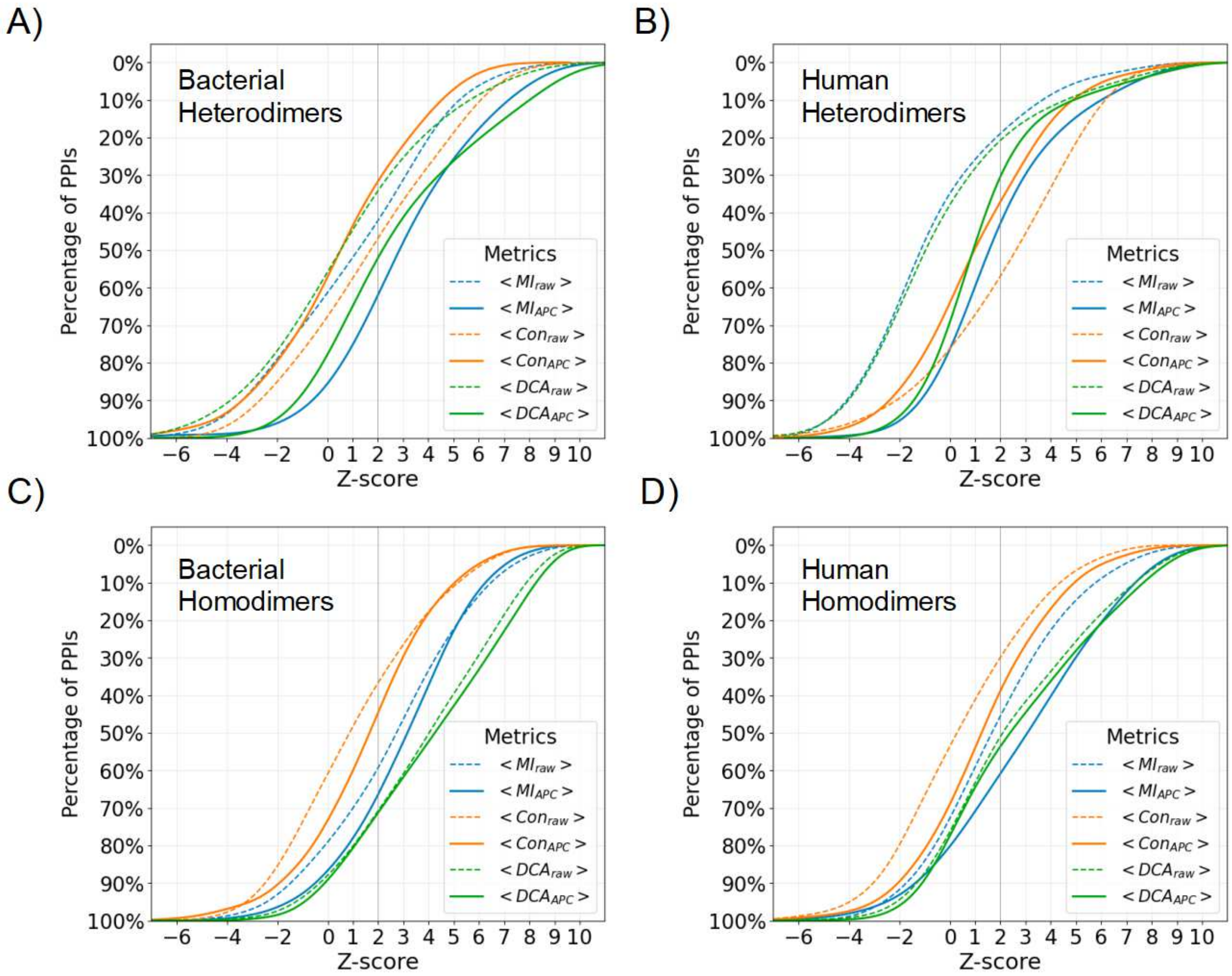

SI Figure S1. Percentage of PDB PPIs as a function of Z-score for raw and APC-corrected metrics averaged over interface contacts. Colors and line type defined in the legend indicate curves for different metrics

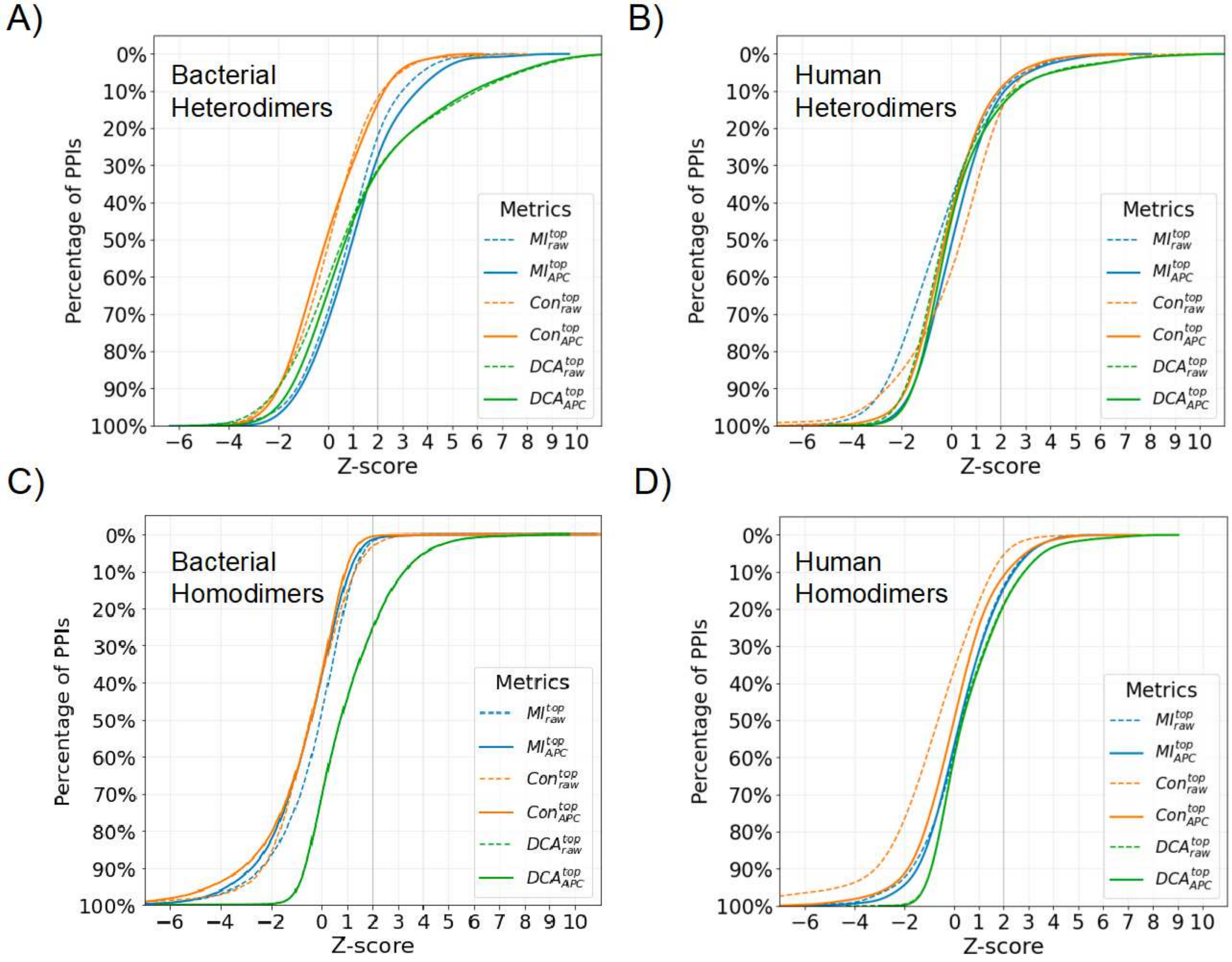

SI Figure S2. Percentage of PDB PPIs as a function of Z-score for the interface contact with the top value for a given metric. Colors and line type defined in the legend indicate curves for different metrics.

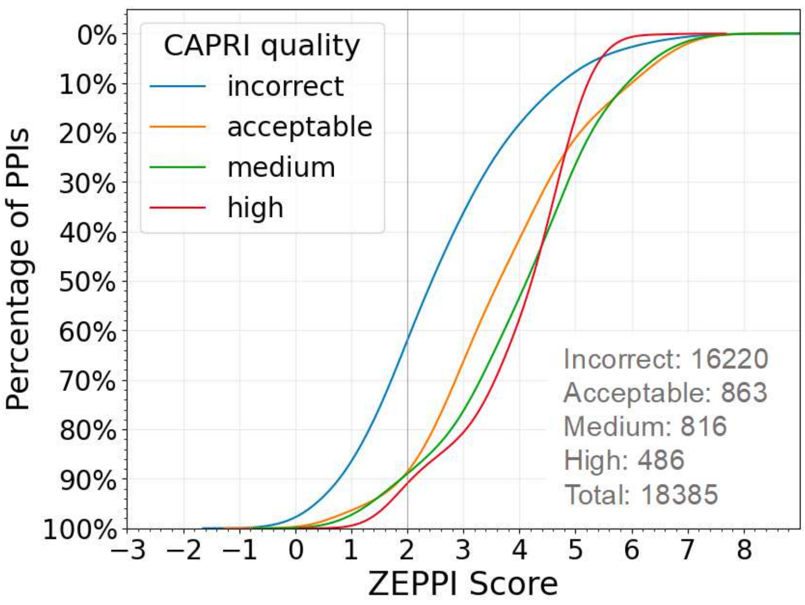

SI Figure S3. Percentage of CAPRI structures and decoys having a given ZEPPI score. Percentages are plotted along the y-axis for four classes of CAPRI models defined in the legend. The total number of models in each class is indicated in the text at the lower left.

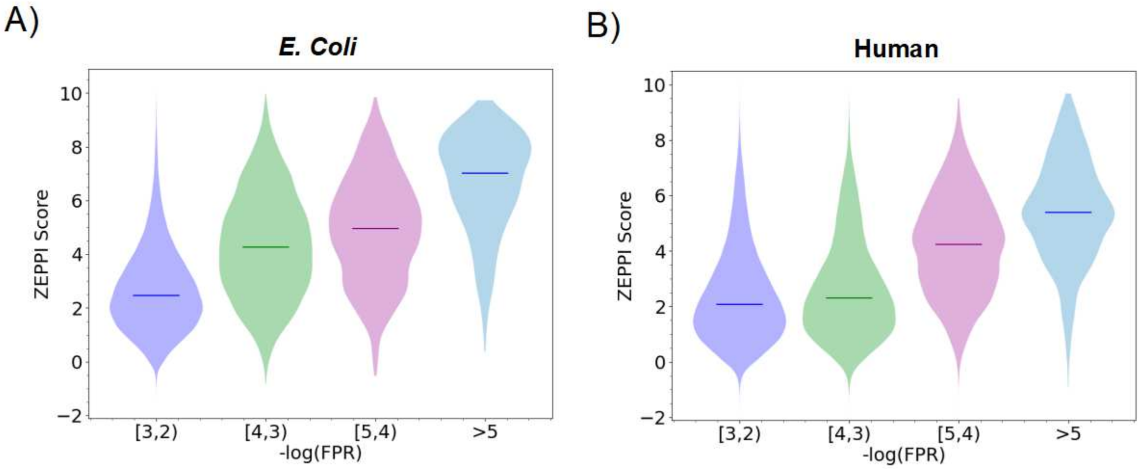

SI Figure S4. Relationship between the ZEPPI score and FPR of PrePPI-predicted PPIs . FPR ranges are indicated below each set of color-coded violin charts, where the median ZEPPI scores are shown as a bar.

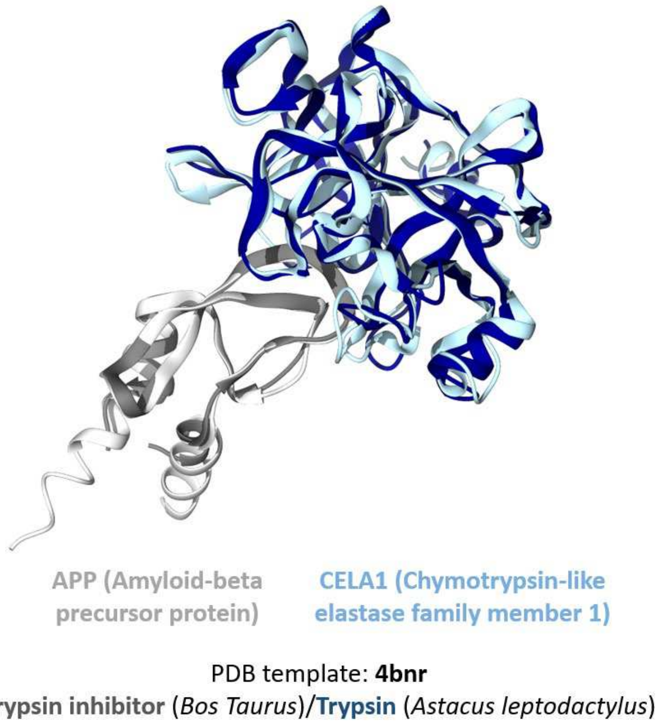

SI Figure S5. Interaction model for Amyloid-beta precursor protein, APP(294-341) (light gray), and Chymotrypsin-like elastase family member 1, CELA1(19-258) (light blue). The template for modeling (PDB ID: 4bnr) is a complex of Trypsin inhibitor from Bos Taurus (dark gray) and Trypsin from Astacus leptodactylus (dark blue). PrePPI FPR < 0.0001, and ZEPPI score = 5.1. The pairwise sequence identities between queries and template chains are 26% and 33%, respectively.

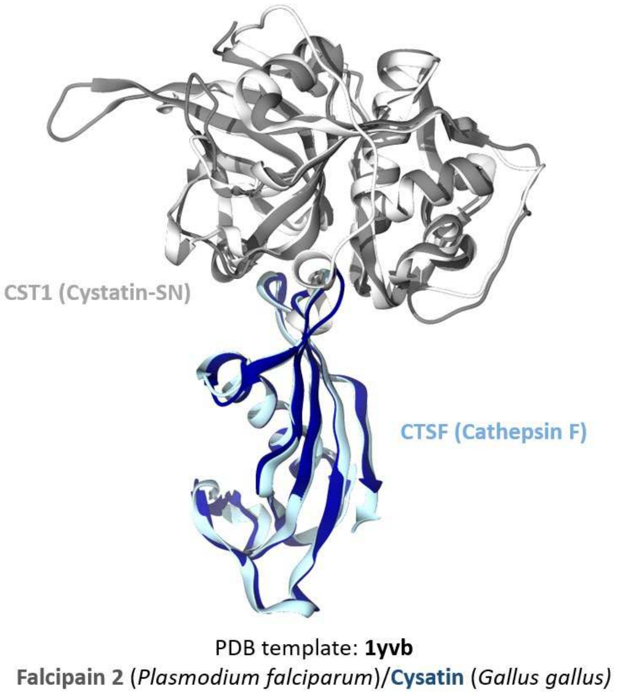

SI Figure S6. Interaction model for Cystatin-SN, CST1(35-141) (light gray), and Cathepsin F, CTSF(244-484) (light blue). The template for modeling (PDB ID: 1yvb) is a complex of Falcipain 2 from Plasmodium falciparum (dark gray) and Cystatin from Gallus gallus (dark blue). PrePPI FPR < 0.005, and ZEPPI score = 5.9. The pairwise sequence identities between queries and template chains are 19% and 32%, respectively.

SI Table S1. Properties and ZEPPI performance of each CAPRI target.

ID	N_{MSA}	$< N_{IFR} >$	$N_{\geq accpt.}$	$N_{incorrect}$	AUROC (*ZEPPI*)
Target29	623	64	167	1916	0.926
Target30	155	42	2	1341	0.550
Target32	62	70	15	584	0.687
Target35	161	65	3	496	0.528
Target37	142	48	99	1401	0.542
Target39	267	58	4	1396	0.843
Target40	55	61	588	1592	0.664
Target41	27	50	371	829	0.755
Target46	1396	56	24	1675	0.651
Target47	24	53	611	440	0.930
Target50	88	3	133	1318	0.734
Target53	2112	55	130	1270	0.742
Target54	198	49	19	1381	0.763

Supplementary Files

This is a list of supplementary files associated with this preprint. Click to download.

- flatHonigepc.pdf
- flatHonigrs.pdf
